# Cytokine Levels and Human Herpesviruses in Saliva from Clinical Periodontal Healthy Subjects with Peri-Implantitis: A Case-Control Study

**DOI:** 10.1155/2018/6020625

**Published:** 2018-08-06

**Authors:** Jaime S. Marques Filho, Jorge Gobara, Gustavo Vargas da Silva Salomao, Laura M. Sumita, Jamil A. Shibli, Renato G. Viana, Humberto O. Schwartz Filho, Claudio Sergio Pannuti, Paulo Henrique Braz-Silva, Debora Pallos

**Affiliations:** ^1^Department of Dentistry, University Santo Amaro, São Paulo, SP, Brazil; ^2^Division of General Pathology, Department of Stomatology, School of Dentistry, University of São Paulo, São Paulo, SP, Brazil; ^3^Department of Virology, Institute of Tropical Medicine of São Paulo, University of São Paulo, São Paulo, SP, Brazil; ^4^Department of Periodontology and Oral Implantology, University of Guarulhos, Guarulhos, SP, Brazil

## Abstract

This study evaluated the presence of cytokines (IL-1*β*, IL-2, IL-4, IL-6, MCP-1, MIP-1*α*, MIP-1*β*, and TNF-*α*) and human herpesvirus (HSV1, HSV2, EBV, CMV, VZV, HHV6, HHV7, and HHV8) in saliva samples taken from subjects with and without peri-implantitis. Forty-two periodontally healthy subjects were divided according to peri-implant condition: healthy and peri-implantitis groups. The clinical parameters as probing depth, clinical attachment level, plaque index, gingival bleeding, bleeding on probing, and suppuration were evaluated. For cytokine detection, multiplex analysis was performed, and PCR assay was used to identify herpesviruses. No significant differences were found in cytokine levels between groups (*p* > 0.05). The presence of herpesvirus was 1.97-fold higher in patients with peri-implantitis (odds ratio, CI 0.52–7.49). The association of the presence or absence of herpesvirus with the salivary markers was statistically significant for MIP-1*β* (*p* = 0.0087) and TNF-*α* (*p* = 0.0437) only in the peri-implantitis group. The presence of herpesviruses in patients with peri-implantitis suggests the development of a proinflammatory environment, which is characterized by increased expression of MIP-1*β* and TNF-*α* in saliva.

## 1. Introduction

Peri-implantitis is an infectious disease that affects the soft and hard peri-implant tissues, leading to bone loss [1–4]. Similar to periodontal diseases, peri-implant diseases are diagnosed after carefully and well-conducted clinical measurements including probing depth, clinical attachment levels, and evaluation of peri-implant bone level alterations over time using standardized radiographies. These procedures are based on clinical training of the professionals as well as their skills. In the last decades, several studies have been conducted to use less invasive and labor-intensive diagnostic tools to detect periodontal and peri-implant diseases [5–8]. In this sense, saliva is a noninvasive, abundant biological fluid, easily collected, and has been used as a diagnostic tool for a variety and range of diseases [9].

Complementarily, the pathogenesis of peri-implantitis presents a well-established correlation between the inflammatory processes and the presence of periodontal bacteria; however, the study of inflammatory microenvironment in association with herpesvirus infection in this process is currently under research. Previous studies suggest that human herpesviruses can play an important role in the pathogenesis of periodontitis [10, 11]. According to the World Health Organization, viruses of the *Herpesviridae* family have infected 90% of the world's population. To date, eight different types of human herpesvirus have been identified: herpes simplex viruses' types 1 and 2, varicella zoster virus, Epstein-Barr virus, cytomegalovirus, roseoloviruses HHV-6 (A and B) and HHV-7, and Kaposi sarcoma-associated herpes virus (HHV-8). Both the Epstein-Barr virus and HHV-8 are carcinogenic [12–14]. Human herpesviruses persist for the lifetime of a host by establishing latent infections, which are broken by periodic reactivation events. In immune-competent individuals, primary infection is rarely symptomatic, and following the establishment of latency, the virus maintains a persistent infection that is effectively controlled by the immune system. These viruses are associated with various oral lesions and have been reported to be present in different types of cells, including B lymphocytes, T lymphocytes, and epithelial cells [15].

In addition, herpesviruses have been associated with periodontal disease, especially cytomegalovirus and Epstein-Barr virus (EBV) [10, 11, 16]. Few studies have shown an association between herpesviruses and peri-implant disease; however, involvement of these viruses in the pathogenesis of peri-implantitis is controversial [17–19]. It has been suggested that herpesviruses possess factors that can trigger periodontal tissue destruction and that periodontal herpesvirus activation can destabilize local host immune responses. Whether herpesviruses possess biological mechanisms that can influence peri-implantitis must be evaluated carefully [17, 20].

Knowing that the viruses can induce the inflammatory process, our hypothesis is that they may play a possible role in peri-implantitis. The hypothesis was to evaluate if a diseased dental implant could impact on cytokine levels according to the presence of target virus. Therefore, the purpose of this case-control study was to compare the levels of cytokines (IL-1*β*, IL-2, IL-4, IL-6, MCP-1, MIP-1*α*, MIP-1*β*, and TNF-α) and the presence of human herpesviruses (HSV-1, HSV-2, EBV, CMV, VZV, HHV6, HHV7, and HHV8) in saliva samples taken from subjects with and without peri-implantitis.

## 2. Materials and Methods

### 2.1. Subject Population

The study protocol was explained to each of the subjects, and signed informed consent was obtained. The Institutional Committee of Ethics in Clinical Research of the University of Guarulhos approved the study protocol according to number 205/03. All the procedures involving human participants were performed in accordance with the institution's and/or national research committee's ethical standards and with the 1964 Helsinki declaration and its later amendments or comparable ethical standards.

Forty-two patients with implant-supported restoration in occlusal function for at least 2 years were enrolled in this case-control study. The patients were divided into 2 groups: the control group (*n* = 21 subjects)—subjects who had at least one healthy dental implant, and the peri-implantitis group (*n* = 21 subjects), subjects with at least one diseased dental implant (peri-implantitis). Briefly, peri-implantitis was characterized by radiographically visible peri-implant bone loss greater than 3 mm, probing depth > 4 mm with bleeding on probing and/or suppuration [21, 22].

Subjects were excluded if they present an implant with a hydroxylapatite-coated surface; had moderate to severe chronic periodontitis (i.e., suppuration, PD > 5 mm and bleeding on probing in more than 30% of subgingival sites); had aggressive periodontitis; had taken antibiotics or anti-inflammatory drugs within 6 months prior to the clinical examination; had received periodontal or peri-implant therapy within 6 months; had a chronic medical disease or condition; presented implant-supported prostheses with mobile abutments and/or screws, as well as fractured prosthetic crowns made of ceramic or resin (to avoid occlusal interference); had clinically detectable mobility of the implant (lack of osseointegration); or smoked tobacco.

### 2.2. Clinical Assessments

Clinical parameters, such as the presence (1) or absence (0) of plaque, gingival bleeding, bleeding on probing, suppuration, and measures of probing epth (mm) and clinical attachment level (mm), were determined at 6 sites per implant by each of the two calibrated examiners.

Two calibrated investigators performed all clinical examination [22, 23]. The interexaminer variability was 0.3 mm for PD and 0.3 mm for CAL. For the first examiner, the intraexaminer mean SE variability was 0.1 mm for PD and 0.1 mm for CAL. The second examiner presented a mean SE variability of 0.20 mm and 0.22 mm for PD and CAL, respectively. The periodontal parameters registered dichotomously, that is, plaque accumulation, gingival bleeding, bleeding on probing, and suppuration, were calculated in the same way, with two different evaluations by the k-light test (*p* < 0.05), which considers the contribution of agreement by chance. The interobserver agreement ranged between 0.85 and 0.95, while the intraobserver agreement was between 0.80 and 0.96 for the first examiner and 0.80 and 0.87 for the second examiner.

### 2.3. Saliva Collection and Laboratory Procedures

Saliva samples were collected at the clinic on a different day than the dental examination; subjects were asked not to consume food or liquids and to refrain from brushing for 12 hours prior to sample collection. Whole saliva was collected, which was accumulated for five minutes, into identified sterilized plastic tubes that were subsequently stored at −20°C. DNA extraction of the saliva samples was performed using a DNA Blood Mini kit QIAamp® kit (QIAGEN Group, Valencia, California, USA) according to directions. The quantification of samples was performed using a spectrophotometer (NanoDrop ND-1000 Spectrophotometer v.3.0.1, Labtrade) following the purity and quality parameters suggested by the manufacturer.

### 2.4. Human Herpesvirus PCR and Restriction Enzyme Digestion

Two primers, HSVP1/P2 and VZVP1/P2, were designed to flank a well-conserved region of the DNA polymerase gene, based on an alignment of DNA sequences from the eight known human herpesviruses as described by Johnson et al. in 2000 [24]. The positive samples were submitted to enzymatic digestion with BamHI and BstUI restriction enzymes (New England Biolabs) for specific determination of each one of the eight herpesviruses, also described in previous works of our group [25–27].

### 2.5. Analysis of Cytokines by Luminex Assay

The concentrations of eight salivary cytokines, including IL-1*β*, IL-2, IL-4, IL-6, MCP-1, MIP-1*α*, MIP-1*β*, and TNF-*α*, were measured. Cytokine levels were determined using a high-sensitivity human cytokine 8-plex by Millipore (Millipore Corporation, Billerica, MA). The Milliplex kits were developed with microspheres and were based on immunoassays.

### 2.6. Data Analysis

Analyses were performed using GraphPad Biostat Prism versions 4.0 and 5.0. A normality test (Kolmogorov-Smirnov) was initially performed, after which comparisons between groups (healthy and peri-implantitis) at each trial were made using Mann–Whitney *U* or Student's *t*-tests. To compare the probability of occurrence of virus in cases of healthy versus peri-implantitis patients, an odds ratio test was employed. A significance level of 5% was used in all analysis.

## 3. Results

A total of 42 patients (21 healthy and 21 peri-implantitis) were included in this study.  Table 1 presents the demographic and clinical data of the subjects from both groups. All clinical parameters were statistically higher in the peri-implantitis group, except for the percentage of sites with plaque (*p* > 0.05).

The distribution of herpesviruses in the study patients is shown in Table 2. In the healthy group, 16 (76.2%) individuals were negative for all herpesviruses, whereas 2 (9.5%) were positive for EBV and HHV7 and 3 (14.3%) were positive for HHV7. The peri-implantitis group included 13 (61.9%) individuals who were negative for all herpesviruses; one (4.7%) who was positive for EBV, HHV6B, and HHV7; 3 (14.3%) who were positive for EBV and HHV7; and four (19.1%) who were positive for HHV7. The odds ratio (95% confidence interval) of the comparison of the groups for positive sites of viral infection was 1.97 (CI 0.52–7.49). Therefore, the presence of virus was 1.97 times higher in patients with peri-implantitis. The herpesviruses CMV, VZV, HSV1, HSV2, and HHV8 were not found in any of the saliva samples.

The salivary markers were not statistically significant between groups as depicted in Table 3. In determining the association of the presence (*n* = 13) or absence (*n* = 29) of virus with salivary markers from both groups (i.e., 42 subjects), a significance positive difference was found in the MIP-1*β* marker in the presence of virus (5.18 ± 3.75) compared to in the absence of virus (2.04 ± 2.15; *p* = 0.0174), as well as in TNF-*α* in the presence of virus (1.87 ± 1.88) compared to in the absence of virus (0.43 ± 0.35, *p* = 0.0120). The remaining markers did not exhibit any differences between the two groups (Table 4).

When the presence or absence of virus with salivary markers was compared, significant differences for MIP-1*β* and TNF-*α* were found only in the peri-implantitis group (Table 5). Additionally, subjects who were virus-positive and either suffering from peri-implantitis or in good health with those who were virus-negative and either suffering from peri-implantitis or in good health, no significant differences between groups were found (Table 6).

## 4. Discussion

In the present study, we analyzed the associations between the presence of herpesvirus and a collection of salivary markers in subjects with and without peri-implantitis. The presence of virus was 1.97-fold higher in subjects with peri-implantitis (odds ratio 1.97, CI 0.52–7.49). EBV presented higher frequency of detection compared with CMV, in agreement with previous data [11, 17, 20, 28, 29]. Few papers in the literature have studied the presence of herpesvirus in relation to peri-implant disease [17–20, 28, 29]. To date, this is the first study that analyzed the presence of all eight types of human herpesviruses (HSV-1, HSV-2, EBV, CMV, VZV, HHV6, HHV7, and HHV8) in the saliva of subjects with peri-implantitis.

In comparing the levels of biomarkers in the saliva, no significant differences were found between the two groups. Similar to our results, Fonseca et al. reported that there were no differences between patients with mucositis and those with peri-implantitis in levels of IL-1*β*, IL-2, IL-4, IL-5, IL-6, IL-7, IL-8, IL-10, IL-12, IFN-*γ*, or TNF-*α* in saliva [30].

In the comparison of salivary markers between patients who were positive and those who were negative for herpesvirus (independent of peri-implant condition), we found significantly higher levels of MIP-1*β* and TNF*α* in the peri-implantitis group. When the salivary markers were analyzed according to healthy or diseased and the presence or absence of herpesvirus, we noted that the peri-implant group produced the same results as the total group; furthermore, healthy subjects exhibited no differences in any of the markers. The same profile was observed when comparing the frequency of positive viral presence between the peri-implantitis and healthy groups and when comparing the frequency of negative viral presence between the peri-implantitis and healthy groups.

A previous review and meta-analysis concluded that inflammatory mediators, such as IL-1*β* and TNF-*α*, could be used as additional diagnostic criteria for peri-implant infections in peri-implant crevicular fluid (PICF) [31]. In our study, we found differences in the levels of MIP-1*β* and TNF-*α* in saliva that were associated with the presence of herpesvirus in the peri-implantitis group only.

In the literature, few papers have measured salivary markers in peri-implantitis patients; most have shown results from PICF [31]. The use of saliva and crevicular fluid to establish biomarker profiles has increased, especially in the diagnosis of periodontal and peri-implant disease [19, 21, 30–34]. Nowzari et al. [18] concluded that when periodontal pathogens were detected, cytokine levels were increased around teeth and implants; however, they did not detect the presence of CMV in crevicular fluid samples from teeth and implants [18]. Jankovic et al. [17] also found a high prevalence of virus (CMV and EBV) in subgingival plaque at peri-implantitis sites, suggesting a possible active pathogenic role in the development of peri-implantitis, comparing healthy, mucositis, and peri-implantitis sites [20].

The present study does not take into account the bacterial detection in crevicular peri-implant sulci; instead, the cytokine levels sometimes should be related with bacterial processes at dental biofilm, either at supra- or at subgingival environment. It could be speculated that the absence of correlation among some cytokines often related with periodontal disease progression and activity and the peri-implantitis status could be influenced by the source of evaluation, in our case, the saliva samples. Marker concentration levels in peri-implant crevicular fluid are higher when compared with saliva, probably due to the dilution at total volume of saliva. However, the presence of herpesviruses being associated with some cytokines in saliva samples demonstrates the interaction between the virus and the oral environment, providing a proinflammatory milieu.

Finally, this study presents an important limitation: the absence of a comparative analysis between intra- and extracrevicular detection of herpesvirus and cytokines. Although recent studies have suggested the similarity and sensibility of the saliva as a detection tool for several host-derived biomarkers and periodontal pathogens, some of these biomarkers could be influenced by the interaction among salivary proteins and therefore expressed in lower levels or could not be detected [5–7]. In this sense, this analysis should be impacted by the sample size, mainly in the association analysis of some markers and the presence or absence of virus detection (Tables 5 and 6).

In conclusion, our findings suggested that the herpesvirus detection could be associated with peri-implantitis and therefore with the development of a proinflammatory environment, similarly as described with bacterial infection, which is characterized by increased expression of MIP-1 and TNF in saliva. Further studies using a large sample population should investigate the presence of these markers in both peri-implant crevicular fluid and saliva samples.

## Figures and Tables

**Table 1:** Mean ± SD of clinical characteristics of the subjects in both groups.

Clinical variables	Healthy	Peri-implantitis	*p*
*n* subjects	21	21	
Age (years)^ns^	49.0 ± 12.2	52.6 ± 12.1	0.3778
Gender (m : f)^ns^	8 : 13	3 : 18	
Probing depth (mm)^∗^	3.27 ± 0.99	5.06 ± 2.28	**0.0050** ^∗^
Clinical attachment level (mm)^∗^	0.15 ± 0.64	5.08 ± 2.03	**<0.0001** ^∗^
% sites with:			
Plaque^ns^	43.1 ± 43.7	31.7 ± 40.1	0.5058
Gingival bleeding^∗^	26.5 ± 35.3	53.9 ± 36.1	**0.0227** ^∗^
Bleeding on probing^∗^	50.0 ± 36.4	80.8 ± 30.8	**0.0089** ^∗^
Suppuration^∗^	0 ± 0	10.7 ± 0.1	**<0.01** ^∗^

Mann–Whitney *U* and chi-square test. ns: nonsignificant. ^∗^Significant.

**Table 2:** Presence (+) or absence (−) of herpesviruses in saliva samples taken from the healthy and peri-implantitis groups. From the total, 16 healthy and 13 peri-implantitis patients are negative for all viruses.

^∗^Patient	EBV	HHV6B	HHV7	CMV, VZV, HSV1, HSV2, HHV8
Healthy				
3	+	−	+	−
9	+	−	+	−
11	−	−	+	−
24	−	−	+	−
32	−	−	+	−
Peri-implantitis				
2	+	−	+	−
12	−	−	+	−
13	−	−	+	−
25	−	−	+	−
27	−	−	+	−
29	+	−	+	−
37	+	−	+	−
39	+	+	+	−

+: positive; −: negative. ^∗^Number of each patient in the study protocol.

**Table 3:** Comparison of salivary marker levels (mean ± standard deviation) between the healthy and peri-implantitis groups. Student's *t*-test and Mann–Whitney test (*p* > 0.05).

	Peri-implantitis	Healthy	*p*
	(*n* = 21 subjects)	(*n* = 21 subjects)	
IL-1*β*	5.31 ± 13.20	1.87 ± 2.01	0.4930
IL-2	0.03 ± 0.04	0.06 ± 0.08	0.3554
IL-4	0.0026 ± 0.0045	0.0173 ± 0.0369	0.5445
IL-6	0.93 ± 1.22	0.82 ± 0.81	0.8326
MCP-1	22.60 ± 19.82	65.64 ± 62.63	0.0539
MIP-1*α*	4.04 ± 3.53	5.00 ± 4.61	0.5431
MIP-1*β*	2.67 ± 2.43	3.53 ± 2.82	0.3268
TNF*α*	1.02 ± 0.86	1.64 ± 1.86	0.9868

**Table 4:** Comparison of salivary marker concentration (mean ± standard deviation) between positive (+) and negative (−) subjects for herpesvirus. Student's *t*-test and Mann–Whitney test (^∗^*p* < 0.05).

	Virus +	Virus −	*p*
	(*n* = 13 subjects)	(*n* = 29 subjects)	
IL-1*β*	16.23 ± 67.54	6.91 ± 13.69	0.7654
IL-2	0.06 ± 0.07	0.03 ± 0.05	0.0810
IL-4	0.018 ± 0.027	0.008 ± 0.02	0.2378
IL-6	0.89 ± 1.00	0.82 ± 1.05	0.8090
MCP-1	30.46 ± 32.91	61.83 ± 57.93	0.1081
MIP-1*α*	5.42 ± 4.50	3.32 ± 3.88	0.1407
MIP-1*β*^∗^	5.18 ± 3.75	2.04 ± 2.15	**0.0174**
TNF*α*^∗^	1.87 ± 1.88	0.43 ± 0.35	**0.0120**

**Table 5:** Comparison of viral frequency (positive + or negative −) between subjects with and without peri-implantitis. Student's *t*-test and Mann–Whitney test (*p* > 0.05).

	Peri-implantitis		*p*	Healthy		*p*
	+	−		+	−	
				(*n* = 5 subjects)	(*n* = 16 subjects)	
	(*n* = 8 subjects)	(*n* = 13 subjects)				
IL-1*β*	4.56 ± 12.41	6.33 ± 15.03	1.0000	1.91 ± 1.98	2.45 ± 2.64	0.6348
IL-2	0.05 ± 0.04	0.02 ± 0.02	0.2924	0.07 ± 0.08	0.05 ± 0.09	0.3980
IL-4	0.003 ± 0.005	0.001 ± 0,003	0.2796	0.016 ± 0.035	0.02 ± 0.04	0.6930
IL-6	0.96 ± 1.27	0.89 ± 1.23	1.0000	0.84 ± 0.79	0.40 ± 0.33	0.6658
MCP-1	22.11 ± 21.21	23.42 ± 19.18	0.9578	71.60 ± 61.38	105.7 ± 79.43	0.7414
MIP-1*α*	4.47 ± 3.58	3.41 ± 3.65	0.4722	5.43 ± 4.59	1.42 ± 0.84	0.1500
MIP-1*β*	3.48 ± 2.35	1.56 ± 2.22	**0.0087** ^∗^	3.81 ± 3.07	2.75 ± 2.05	0.4865
TNF*α*	1.28 ± 0.98	0.50 ± 0.45	**0.0437** ^∗^	1.99 ± 2.05	0.33 ± 0.14	0.2256

^∗^Statistic significant.

**Table 6:** Comparison of the frequency of virus-positive (+) subjects and virus-negative (−) subjects between the peri-implantitis and healthy groups. Student's *t*-test and Mann–Whitney test (*p* > 0.05).

	Virus (+)		*p*	Virus (−)		*p*
	Peri-implantitis	Healthy		Peri-implantitis	Healthy	
IL-1*β*	4.56 ± 12.41	1.91 ± 1.98	0.4751	6.33 ± 15.03	2.45 ± 2.64	0.7688
IL-2	0.05 ± 0.04	0.07 ± 0.08	0.6296	0.02 ± 0.02	0.05 ± 0.09	0.8143
IL-4	0.003 ± 0.005	0.016 ± 0.035	0.4325	0.001 ± 0.003	0.02 ± 0.04	0.7270
IL-6	0.96 ± 1.27	0.84 ± 0.79	0.8625	0.89 ± 1.23	0.40 ± 0.33	1.0000
MCP-1	22.11 ± 21.21	71.60 ± 61.38	0.3390	23.42 ± 19.18	105.7 ± 79.43	0.8114
MIP-1*α*	4.47 ± 3.58	5.43 ± 4.59	0.9444	3.41 ± 3.65	1.42 ± 0.84	0.9148
MIP-1*β*	3.48 ± 2.35	3.81 ± 3.07	0.4264	1.56 ± 2.22	2.75 ± 2.05	0.1213
TNF*α*	1.28 ± 0.98	1.99 ± 2.05	0.5856	0.50 ± 0.45	0.33 ± 0.14	0.8002

## Data Availability

The data used to support the findings of this study are available from the corresponding author upon request.
